# Stem cell-based therapy for ameliorating intrauterine adhesion and endometrium injury

**DOI:** 10.1186/s13287-021-02620-2

**Published:** 2021-10-30

**Authors:** Yu-Ting Song, Peng-Cheng Liu, Jie Tan, Chen-Yu Zou, Qian-Jin Li, Jesse Li-Ling, Hui-Qi Xie

**Affiliations:** 1 Laboratory of Stem Cell and Tissue Engineering, Orthopedic Research Institute, Med-X Center for Materials, State Key Laboratory of Biotherapy, West China Hospital, Sichuan University, Chengdu, 610041 China; 2 Department of Medical Genetics, West China Second Hospital, Sichuan University, Chengdu, 610041 China

**Keywords:** Intrauterine adhesion, Endometrium regeneration, Stem cells, Regenerative medicine

## Abstract

Intrauterine adhesion refers to endometrial repair disorders which are usually caused by uterine injury and may lead to a series of complications such as abnormal menstrual bleeding, recurrent abortion and secondary infertility. At present, therapeutic approaches to intrauterine adhesion are limited due to the lack of effective methods to promote regeneration following severe endometrial injury. Therefore, to develop new methods to prevent endometrial injury and intrauterine adhesion has become an urgent need. For severely damaged endometrium, the loss of stem cells in the endometrium may affect its regeneration. This article aimed to discuss the characteristics of various stem cells and their applications for uterine tissue regeneration.

## Introduction

In 1948, Joseph Asherman first described the diagnosis, anatomy, etiology, prophylaxis, therapy and complication of a specific type of amenorrhea, which was later referred as Asherman’s syndrome (AS) [1], a condition also known as intrauterine adhesion (IUA).

IUA refers to the complication due to damage of endometrial basal layer, for which mechanical trauma, infection and other factors may be attributable. The presence of adhesion tissue may lead to partial or complete obliteration of the uterine cavity and/or cervical canal [2], which may consequently result in deformation or even disappearance of the uterine cavity [3]. Under normal conditions, the endometrial functional layer is periodically shed under hormone regulation, and the basal layers are important for the repair and regeneration of the surface. Therefore, disturbances to the structures of basal layers, such as repair disorder, may exacerbate this process and lead to the occurrence of IUA [3].

## Formation of adhesion

IUA may be classified as primary adhesion after pregnancy-related curettage or hysteroscopic surgery, as well as secondary adhesion reoccurred after adhesiolysis [4]. The European Medicine Agency (EMA) has estimated the prevalence of AS to be 0.04% [5]. More than 90% of IUA are related to pregnancy [6]. It is usually developed following the manipulation of early abortion or postpartum-related curettage, and may be considered as a postoperative complication of intrauterine surgery [2, 7, 8]. Uterine trauma and postoperative infection, including abdominal myomectomy, cervical biopsy or polylectomy, and insertion of intrauterine device (IUD), are also common causes of IUA [7]. Moreover, reproductive system infection, which may be due to non-pregnancy uterine cavity trauma and congenital uterine malformation, is another risk factor of IUA [9]. Additionally, IUA may also be correlated with irregular uterine dilation, inadequate disinfection, unsoftened cervix, long forceps scraping time or high intraoperative uterine negative pressure.

IUA can cause severe endometrial dysfunctions including infertility and menstrual disorders such as periodic hypogastralgia, hypomenorrhea, amenorrhea, endometriosis, recurrent pregnancy loss, and secondary infertility [10]. Recurrent miscarriage is associated with IUA too. Approximately 20–30% of patients with recurrent pregnancy loss suffer from IUA [11–14]. However, it is still uncertain whether IUA is a cause or consequence of recurrent miscarriage [6]. Severe IUA is usually accompanied by endometrial atrophy, which may interfere with embryonic implantation and fetoplacental growth, or even cause infertility [15]. The incidence of IUA among infertile patients is 13% [7], while 8% of infertility cases are secondary to IUA [8].

## Pathological mechanisms

The uterus is formed by a mixture of endometrial, muscular and connective tissues. Histologically, the myometrium in IUA resembles that of the normal myometrium, but usually with increased thickness [16]. The connective tissue formed by thin collagen bundles often derives from dense fibrous strips [5]. Endometrial fibrosis is the common pathological manifestation of IUA, where fibrin is the mainly contributor to the formulation of tissue bridges between the walls of uterine cavity [8]. As damaged endometrium cannot be properly repaired, endometrial stromata will be largely replaced by fibrous tissues, glands and inactive cubocolumnar epithelia which is non-responsive to hormonal stimulation [16, 17]. Under such circumstance, the distinction between the functional and basal layers of the endometrium will be lost.

Endometrial trauma may affect the vascularity of endometrium. As illustrated by pelvic angiography, the distribution of vascularity in endometrium and myometrium may be impaired by traumatic endometrial damages [17]. As indicated by previous study, new blood vessels derived from pre-existing vasculature and vasculogenesis plays a pivotal role in the menstrual cycle [18]. However, the newly regenerated fibrotic tissue is usually without vessels and, concomitantly, the paucity of blood supply in IUA patients may also indicate that uterine artery damage is with fibrosis [7, 17]. Previous studies have shown aberrant activation of fibrosis to be closely associated with pathological changes of IUA [19–22]. Normal wound healing is regulated by a series of complex pro-fibrosis and anti-fibrosis processes [23]. However, excessive endometrial fibrosis may be related to the failure of normal wound healing process, which may aggravate the formation of IUA. Notably, inflammatory response, which occurs following endometrial trauma and may activate down-stream detrimental pathways, is also associated with the process of fibrosis [24].

## Classification

It is necessary to classify IUA in order to summarize their prognosis and correspondingly therapeutic outcome. There are different criteria for the classification of IUA since Asherman original description. Table 1 summarizes the current classification systems and their key features.

**Table 1:** Classification of IUA

Source	Summary of classification	References
Toaff and Ballas	Split into four grades according to the lesion location and size	[25]
March et al.	Classified as minimal, moderate, or severe based on the degree of uterine cavity involvement by HSG	[11]
American fertility society	Complex scoring system of mild, moderate, or severe IUAs based on the extent of cavity obliteration, type of adhesion, and menstrual pattern according to hysteroscopic or HSG assessment	[26]
Valle and Sciarra	Adhesions classified as mild, moderate, or severe according to the extent of uterine cavity occlusion (partial or total) and the type of adhesions by HSG	[27]
Nasr et al.	Creates a prognostic score by menstrual patterns, reproductive performance and hysteroscopy as parameters	[28]

In 1978, Toaff and Ballas first classified IUA based on the result of hysterosalpingography (HSG) [25]. They were classified into four grades according to the location and size of the lesion. In the same year, March et al. proposed a hysteroscopic classification of IUA based on the proportion of uterine cavity shown by HSG, which was classified into 3 grades from mild to severe [11]. The method is relatively simple and is still in use today. Nevertheless, based on the extent of the disease, menstrual pattern, and morphological feature of the adhesions, the American Fertility Society had designed a new scoring system in 1988, which could be used in the hysteroscopy and HSG [26], and added menstrual patterns to the rating parameters for the first time. Moreover, they suggested that the location of adhesion may be important for infertile women, so it is necessary to chart the location and extent of adhesions. In the same year, Valle and Sciarra described a new classification based on the extent of uterine cavity occlusion and type of adhesions [27]. Mild adhesions are filmy and composed of endometrial tissue, moderate adhesions are fibromuscular, while severe adhesion or complete occlusion of the uterine cavity are only composed of dense connective tissue, which had the poorest prognosis. Moreover, they concluded that although almost all patients treated with hysteroscopy were able to recover with normal menstruation, reproductive outcomes parallel the severity of the adhesions.

In 2000, Nasr et al. proposed a new classification method to score IUA using menstrual patterns, reproductive performance and hysteroscopy as parameters [28]. It is the first classification that correlates menstrual pattern and reproductive performance with the prognosis of hysteroscopic adhesiolysis, and they believed that the prognosis depends more upon the type of adhesions and the extent of coverage of tubal ostia. In their proposed rating system, grades I and III were consistent with those of March et al. [11], but for moderate IUAs (grade II), there was an overlap between the two systems (with a sensitivity of 58.3%), which may be related to menstrual and reproductive history of patients.

In 2015, the Society of Obstetrics and Gynecology of Chinese Medical Association proposed a new rating scale based on the previous classification. Compared with the previous rating, the table added the previous history of curettage. However, there is still no consensus over the optimum classification of IUA. Further research is needed, particularly for prediction of reproductive prognosis [6, 9].

## Current treatment options

Currently, there has been no specific guideline for the treatment of IUA. Various therapeutic approaches have been developed for the repair of endometrial injury and prevention of recurrent adhesions. The most common strategy is transcervical resection of the adhesions (TCRA). Hysteroscopic treatment enables lysis of IUAs under direct vision with magnification, in which only blunt dissection is performed at the tip of the hysteroscope [29, 30]. Daniel et al. have found that hysteroscopic resection could significantly reduce the incidence of IUA and increase the rate of pregnancy [31].

However, postoperative outcome may vary as the severity of adhesion is diverse and disparate [32]. The more severe the adhesions are, the more difficult dissection and greater risk of the complications will be. According to some studies, the pregnancy rate of patients with mild IUA was close to 95%, while in the severe group the rate was decreased to 60% after hysteroscopic treatment and subsequent miscarriages percentage could be as high as 75% [14].

In addition to hysteroscopic treatment, hyaluronic acid gel [33], hormone therapy [34], uterine perfusion [35], and amniotic membrane transplantation [36] have also been used for the treatment of IUA. However, such treatments are only applicable to patients with mild and moderate IUA. Therefore, to develop new methods to prevent endometrial injury and manage IUA has become a major demand.

In 2004, the first evidence for the existence of endometrial stem/progenitor cells in the endometrium was reported [37]. Some researchers hypothesized that endometrial repair disorders may be related to local stem cell damage and loss as regeneration and repairing of the endometrium are closely related with stem cells in the endometrium. Thereby, application of stem cells to treat endometrial injury may be an effective strategy to restore endometrial receptivity. This idea outperforms the conventional treatment and opens a new avenue for the treatment of IUA.

## Stem cell therapy

**Fig. 1:**
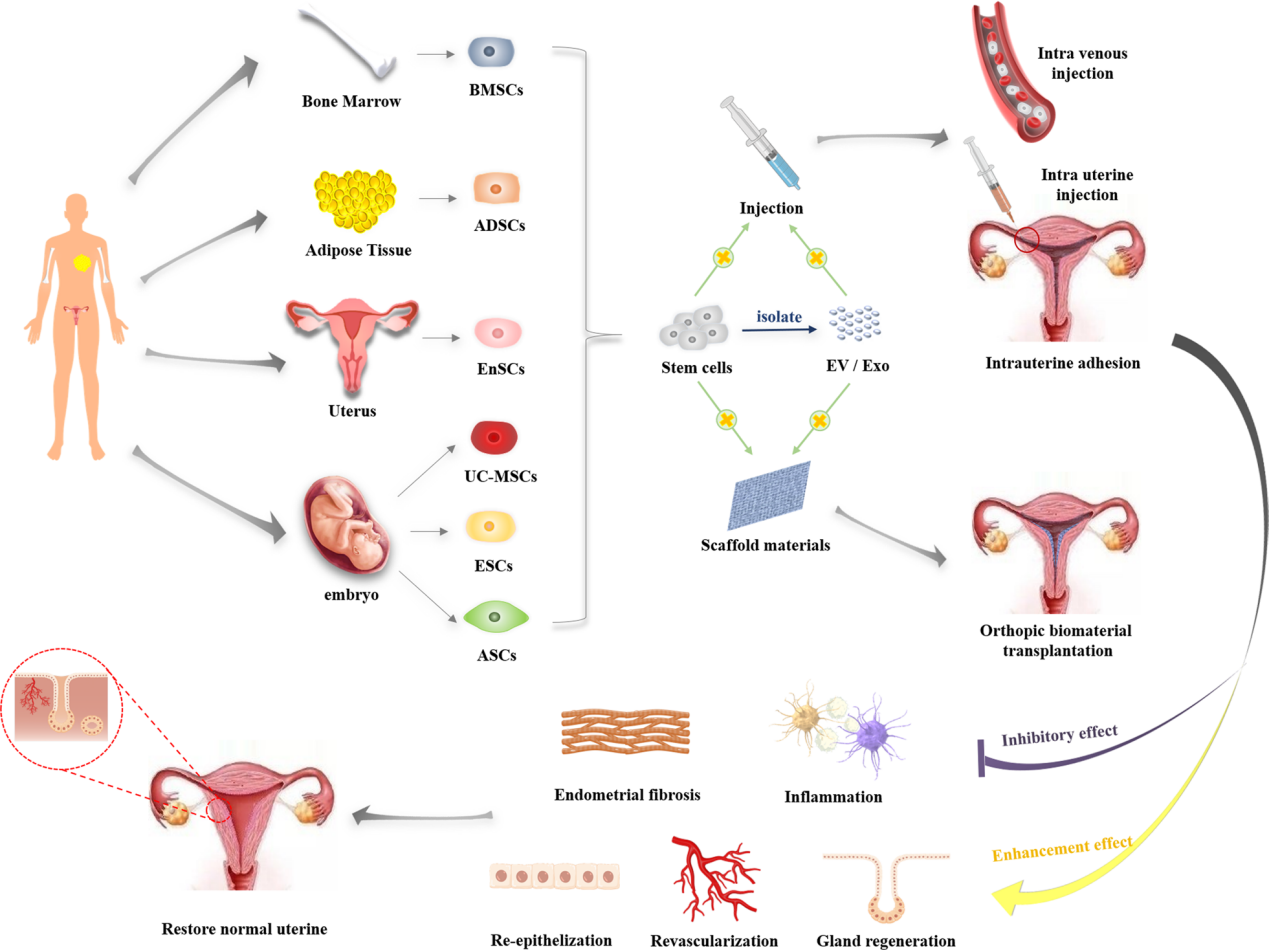
Stem cell-based approaches to uterus regeneration include (1) intravenous stem cells or EV/ Exo, (2) intrauterine injection stem cells or EV/Exo and (3) fabrication of synthetic graft by encapsulating stem cell or EV/Exo therapeutics with biomaterials. After transplantation, stem cells can stimulate the angiogenesis, epithelization and gland regeneration while inhibitory inflammation and endometrial fibrosis, and eventually restore normal uterine structure and function

In 1978, Prianishnikov introduced endometrial stem cells (EnSCs) for the first time [38]. It was not until 2004 that Chan et al. [37] ultimately isolated EnSCs from endometrial tissue, since then the exploration has mounted. Gargett et al. [39, 40] have confirmed the presence of a small number of stromal stem cells and epithelial stem cells in the uterus which could promote endometrial proliferation during the menstrual cycle but were decreased with uterine injury. Such cells were thought to be responsible for the periodic regeneration of the endometrium. Many researchers believe that the homing and migration of stem cells towards the site of lesion plays an important role in tissue regeneration [41–43]. However, there are studies suggesting that the migration and invasion capacities of the stem cells were significantly lower from women with IUA compared with healthy women, which may indirectly affect the self-repairing ability of injured endometrium [44]. Furthermore, when the endometrium is severely damaged, the decrease or loss of stem cells in the endometrium may also affect the regeneration of the endometrium. On the other hand, autologous and allogeneic stem cell transplantation may both be effective for the treatment of IUA [45] (Fig. 1).

With the potential for self-renewal and multi-directional differentiation, stem cells have a broad prospect for the treatment of tissue injury involving the uterine cavity. In recent years, researchers have become focused on the effect of stem cells for the treatment of IUA (Table 2).

**Table 2:** Some resources and features of stem cells which contribute in endometrium regeneration

Stem cell	Major source	Properties	References
BMSCs	Bone marrow	Multi-potent, highly proliferative, good migration ability, self-renewing, immunomodulatory properties	[46, 47]
Autologous adult stem cells	Bone marrow	Donor-derived bone marrow cells have been identified in human uterine endometrium (both stromal and epithelial cells were derived from bone marrow origin). It is unknown whether these cells originate from bone marrow mesenchymal stem cells or circulating endometrial cells originally derived from the endometrium and harbored in bone marrow	[48]
eMSCs	Endometrium	Multi-potent, highly proliferative, self-renewing; coexpression of CD140b (PDGFR-β) and CD146, or expression perivascular markers SUSD2 (W5C5 antibody) SUSD2	[49–51]
ESP	Endometrium	Heterogeneous, presumably containing stem/progenitor cells of each endometrial cell lineage; produce endometrial endothelial, epithelial, and stromal cells in vitro and in vivo	[49, 52, 53]
UC-MSCs	Umbilical cord	Derived from the mesoderm in early development; low immunogenicity; multi-potent cells; high self-renewal ability; multi-differentiation; high proliferative potential	[54–56]
ADSCs	Adipose tissue	Abundant sources; easy sampling; self-renewal; multi-potential differentiation; strong proliferation ability	[57–61]
ESCs	Embryo	Pluripotent stem cells derived from the inner cell mass of a blastocyst; high telomerase activity; significant long-term proliferation potential	[62, 63]
ASCs	Amniotic membrane	Inflammatory suppression, angiogenesis promotion, anti-oxidative stress	[64, 69]

## Bone marrow stem cells (BMSCs)

Bone marrow mesenchymal stem cells (BM-MSCs) are easy to culture in vitro, which makes it the most widely used seed cells for stem cell transplantation. BM-MSCs can differentiate into a variety of non-hematopoietic cells including skeletal myoblasts, cardiac myoblasts, skin epithelia, as well as endothelial, renal, hepatic, and lung cells [32]. Compared with endometrial stem cells, BM-MSCs have greater migration ability, which may lead deposition of a higher proportion of donor cells in vitro [46]. Therefore, BM-MSCs has become a promising candidate seeding cells for repairing uterine damage owing to their merits.

In 2008, Mints et al. [70] have detected presence of Y chromosome in the endometrial cells of a woman who received bone marrow transplants from a male donor. Subsequent studies also illustrated that endometrial angiogenesis was not only from local endothelial cells, but may also come from BMSCs. Cervelló et al. [71] also confirmed the presence of XY donor-derived bone marrow cells in the endometrium of women receiving male bone marrow transplants, and such cells may be an exogenous source of endometrial trans-differentiation cells.

Although uterine damage was localized, BMSCs could migrate to both sides of the uterus, possibly due to the secretion of particular chemokines [10]. Accordingly, secretion of such signal molecules may be an important mechanism for stem cell therapy for uterine cavity injury. Cervelló et al. [72] found that transplanted CD133(+) BMSC have located around endometrial vessels and could induce proliferation of surrounding cells by regulating paracrine factors such as thrombospondin 1 and insulin-like growth factor 1. In addition, the expression of leukemic inhibition factor (LIF), an endometrial receptivity marker in the regenerated endometrium, may also in part contribute to the improved reproductive outcome in a rat model for IUA [73].

Studies have suggested that BMSCs transplantation may repair the damaged endometrium by promoting the expression of ER and PR. Wang et al. [74] injected BMSC into IUA rats by uterine and vein, and found that the BMSCs were more abundant in the uterine injection group after 2 weeks. Interestingly, another study comparing the two methods of injection by using green fluorescent protein (GFP)-expressing BMDCs has found that systemic route of administration could result in better recruitment of BMDCs to the injured uterus after 2–3 weeks [46]. Other studies also found that the implantation and conception rates of IUA rats receiving vein injection of BMSCs were comparable to those with normal uteri, while all untreated IUA rats had failed to conceive [73]. Hence, both the systemic route of administration and local injection could rapidly promote formation of new endometrial glands with subsequently replacing fibrotic scars by increasing ER and PR expression [74]. And, strikingly, fluctuations in systemic hormone levels had no effect on the migration of BMSCs [32].

Clinically, BMSCs can improve the reproductive outcome of IUA patients. Nagori et al. [48] discovered that angiogenic stem cells derived from autologous bone marrow derived stem cells could regenerate injured endometrium and lead to successful pregnancy and delivery. In 2011, a woman with severe IUA has received transvaginal injection of autologous bone marrow stem cells, and after a period of time, her endometrial thickness and blood vessel richness gradually increased, allowing her to eventually maintain embryo growth [48]. In 2016, Santamaria et al. [75] used CD133^+^ BMSCs in conjunction with hormonal replacement therapy for IUA. After 2 months of stem cell therapy, endometrial thickness has increased in 11 IUA patients, with simultaneous amplification of endometrial vascular density and duration and intensity of the menstrual cycle in the first 3 months which returned to the original level after 6 months. And three of them eventually conceive naturally.

Some researchers also reported that implantation of combination of BMSCs with scaffold materials into the IUA uterus was feasible. Zhao et al. [76] recellularized a collagen scaffold with high-density BMSCs and implanted them into the uterus of IUA patients, and found that it could reverse endometrial fibrosis and promote endometrial regeneration by down-regulating the expression of Np63. After the treatment, all five patients successfully attained pregnancies and delivered. Collagen/BMSCs system could enhance proliferation of endometrium and muscular cells, facilitate microvasculature regeneration, and restore the function of endometrium to eventually receive the embryos [77]. Recently, it was found that exosomes secreted by BMSCs could transfer miR-340 to endometriotic stromal cells and effectively attenuate endometrial fibrosis [78]. In addition to collagen scaffolds, other polymer scaffolds combined with BMSC were also exploited for endometrial repair. Xiao et al. [79] constructed a BMSC-loaded elastic poly (glycerol sebacate) (PGS) scaffold, within which the BMSCs could be directly differentiated into endometrial stromal cells after transplantation. Moreover, compared with collagen scaffolds, PGS/BMSC also significantly prolonged the retention time of BMSCs in a rat model for uterine injury.

## Endometrial stem/progenitor cells (EnSCs)

**Fig. 2:**
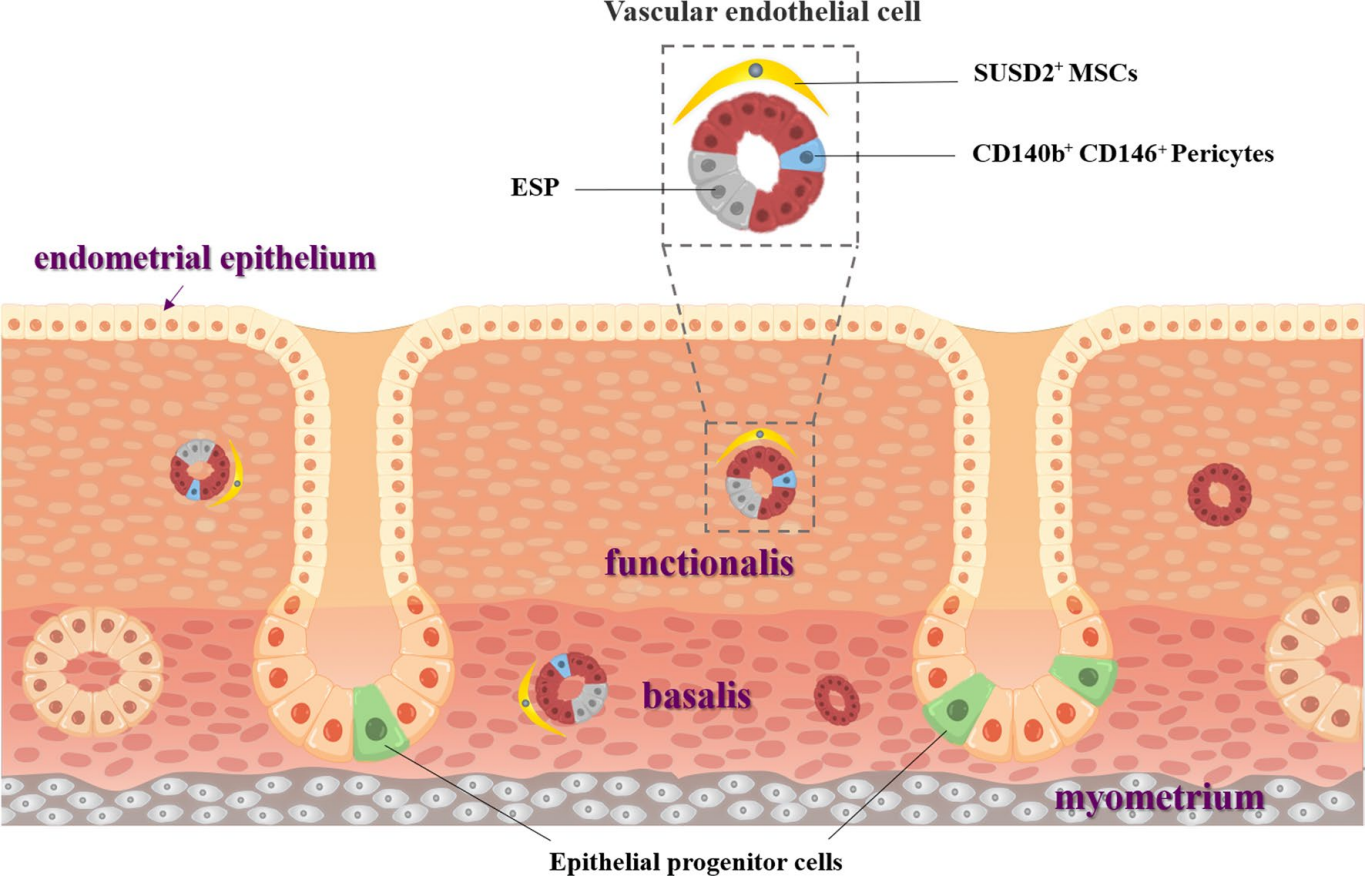
Stem/progenitor cells identified in the human endometrium. (1) Endometrium is composed of endometrial epithelium, functionalis and basalis; (2) Epithelial progenitor cells are postulated to be located in the base of the glands in the basalis; (3) Perivascular SUSD2^+^ (W5C5 antibody) cells with in vitro and in vivo mesenchymal stem/stromal cells (MSCs) properties are found in basalis and functionalis; (4) PDGFR-β/CD140b+CD146+ endometrial MSCs (eMSCs) are pericytes. ESP cells consist of CD31+ endothelial cells and CD140b^+^CD146^+^ pericytes. Adapted from Gurung et al. [85]

Many experiments have confirmed the presence of endometrial stem/progenitor cells, including epithelial stem cells, endometrial mesenchymal stem cells (eMSCs), endothelial progenitor cells (EPCs), which could be activated during the menstrual cycle and conducive to rapid endometrial regeneration following menstruation (Fig. 2). During the past a few decades, the application of EnSCs in uterine regeneration has been rapidly growing for reasons such as high homology with uterine tissue and ease to acquire [80]. Angiogenesis is one of the key steps in endometrial repair. Restructuring and maturation of the vascular network can facilitate embryo implantation [81]. Blood vessel walls are considered as stem cell-niche with a large reservoir of progenitor cells [82]. Previous study has found that eMSCs in the basalis and functionalis are mainly perivascular cells, including CD146^+^ and CD140b^+^ (platelet-derived growth factor-β, PDGFR-β +) pericytes [50, 51], sushi domain-containing-2^+^(SUSD2^+^) cells [83] and CD34^+^ adventitial cells (located in the outermost layer of blood vessels and mainly in the basal layer) [82].

Although both CD34^+^ adventitial cells and CD146^+^ pericytes showed MSCs phenotypes in vitro, they have exhibited a limited potential to regenerate the endometrium. Other studies have shown that CD146^+^CD140b^+^ cells could promote endometrial angiogenesis in rats and capillary formation of HUVECs in vitro [44, 84]. eMSCs have also been found to play certain role in regenerating the endometrial stroma in vivo [46].

EPCs are postulated to reside in the glands of the basalis [86], while endometrial endothelial progenitor cells are recognized as side population cells (SP cells) [50]. As one of the potential endometrial stem/progenitor populations, endometrial side population (ESP) was first identified in short-term culture of endometrial cells [53]. ESP cells can generate endometrial endothelial, epithelial, and stromal cells in vitro and in vivo, which are located both in the basalis and functionalis [49]. Cervelló et al. [52] transplanted hESP beneath the renal capsule of NOD-SCID mice, and found that it could generate human endometrium.

As analogous to other stem cells, EnSCs also have the advantages of self-renewal, multi-differentiation, and high proliferation potential. Such cells can be obtained by scratching the endometrium or isolated from menstrual blood. Such characteristics have made it a potential resource for the treatment of IUA.

Zhang et al. [87] transplanted EnSCs derived from menstrual blood into IUA mice, and found that the endometrial thickness and microvascular density were increased, and the repair of damaged uterus was appreciably accelerated. As reported, EMSCs conditioned medium could activate AKT and ERK pathways, induce overexpression of eNOS, VEGFA, VEGFR1, VEGFR2 and TIE2 in HUVECs, while decrease H_2_O_2_-induced apoptosis of human umbilical vein endothelial cells (HUVECs). In a subsequent study, they found that EnSCs could inhibit myofibroblast activation, resulting in rapid proliferation of epithelial stem cells, which ultimately promoted endometrial wound healing [3]. Moreover, they delineated that Hippo/TAZ is the key signal of EnSCs against the process of endometrial interstitial fibrosis [3]. Another group has also found that EnSCs could substantially accelerate the repair of endometrial damage, in which a paracrine effect and activation of Hippo signaling pathway were validated and the effect was found to be even better when combined with the application of platelet-rich plasma (PRP) [88]. Lin et al. [89] reported that Gli2 signaling could also promote endometrial fibrosis by reducing the level of Gli2 protein in EnSCs conditioned medium (EnSCs-CM) and reduce endometrial fibrosis, a process which was critical in blocking the cell cycle of epithelial stem cells through granulocyte-colony stimulating factor (G-CSF).

In 2016, EnSCs have been approved for usage in clinical trials for endometrial regeneration in patients with IUA. Endometrial thickness of all subjects has significantly increased, and some women even became pregnant following frozen embryo transplantation [90]. Furthermore, a clinical trial using autologous menstrual blood-derived stromal cells for treating severe IUA also showed satisfactory results [90].

In recent years, more researches have focused on the paracrine pathway mediated by stem cells. Similar to other MSCs, EnSCs have been reported to mediate IUA repair through paracrine action of extracellular vesicles (EVs) [80]. A recent study has shown that EnSCs-EV have exerted their immunomodulatory function by inhibiting the activation of CD4^+^ T cells [91].

## Umbilical cord-derived mesenchymal stromal cells (UC-MSCs)

Umbilical cord-derived mesenchymal stromal cells (UC-MSCs) are multi-potent cells with strong self-renewal ability and multi-differentiation potential. Such cells are derived from the mesoderm in early development and have the advantages of easy collection, low immunogenicity, and high proliferative potential [54, 56].

In recent years, some researchers have reported that UC-MSCs could enhance endometrial cell proliferation and vascular remodeling while inhibit excessive fibrosis and inflammation, thereby repair the damaged endometrium and restore fertility [92]. Zheng et al. [93] injected hUC-MSCs into SD rats and showed that it has the ability to differentiate into epithelial cells, vascular endothelial cells, and estrogen receptor cells, which are essential for the supply of blood vessels and interrupting the formation of fibers. All of these could contribute to the restoration of fertility in IUA rats. They also transplanted CM-Dil-labeled hUC-MSCs into rats and found that the hUC-MSCs were not evenly distributed in uterine tissues. More cells had migrated into the stroma and myometrium regions, while almost no cells reached the epithelium of endometrium and gland [93]. They speculated that this may be due to the fact that stroma and myometrium contained more blood vessels than did epithelium, which was in keeping with the fact that MSCs are mainly distributed along the blood vessels [94]. A study has shown that EV could enhance angiogenic processes in endothelial cells [95]. EV derived from hUC-MSCs (hUCMSCs-EV) can also be used as a therapeutic agent for IUA, and was more effective when combined with estrogen [96].

Transplanting scaffolds loaded with UC-MSCs has been investigated as well. Xu et al. [97] constructed a collagen scaffold (CS) loaded with UC-MSCs, and noted that this complex could facilitate the degradation of collagen of uterine scar by upregulating MMP-9 secreted by UC-MSCs, which was instrumental for the repair and regeneration of endometrium, myometrium, and blood vessels. Xin et al. [54] also transplanted CS/UC-MSCs into a model for endometrial damage and discovered that it could maintain the normal luminal structure, promote endometrial regeneration and collagen remodeling, and also increase the expression of estrogen receptors and progesterone receptors. Recent studies have shown that transplantation of UC-MSCs and auto-crosslinked hyaluronic acid (HA) gel might have a dual repair effect with an anti-adhesive property and promotion of endometrial regeneration [98]. By implanting this complex to a rhesus monkey model for IUA, they found that UC-MSCs/HA-GEL was superior to HA-GEL in repairing IUA caused by mechanical injury.

A phase I clinical trial also confirmed that transplantation of UC-MSCs with biodegradable collagen scaffolds into the uterine cavity (by adhesion separation) in patients with recurrent IUA was effective [99]. Three months after the operation, the average and maximum endometrial thickness had both increased, while the IUA score was decreased. At the end of 30-month follow-up period, 10 of the 26 patients attained pregnancy and 8 of them had delivered without compelling birth defects or placental complications.

## Adipose-derived stem cells (ADSCs)

Adipose-derived stem cells (ADSCs) are also a type of mesenchymal stem cells derived from the mesoderm but mainly exist in adipose tissue. Their typical or specific cell markers include CD90(+), CD73(+), CD105 (+), CD45(−) and CD34(−) [57]. At present, ADSCs are one of the most advantageous and extensively researched adult stem cells for cell therapy and tissue engineering. ADSCs have the advantages of abundant source, easy sampling, capable of self-renewal, multi-potential differentiation, as well as strong proliferation ability, and can be obtained from patients themselves thereby avoid ethical problems [59–61]. However, literature on the application of ADSCs for the treatment or prevention of IUA is still scarce. Shao et al. [100] injected green fluorescent protein (GFP)-labelled ADSCs into IUA rats and found that ADSCs could differentiate into endometrial epithelial cells. At 30 days after transplantation, the damaged endometrium was robustly improved, with increased microvascular density, endometrial thickness and glands. The expression of oestrogen and progesterone receptors was also increased. In addition, the fertility of rats was also recovered to some extent. Sun et al. [101] exploited the ADSCs as seed cells to form scaffold-free cell plates with massively retaining of extracellular matrix proteins, growth factors, and a large number of cytokines without enzymolysis [102], and found that ADSCs mainly appeared in the basal layer of the regenerating endometrium at 21 days after transplantation, with some ADSCs differentiated into stromal-like cells.

It has been reported recently that acellular human amniotic membrane (AHAM) can substantially improve the expression of ADSCs angiogenic factors in vitro, and in vivo experiments also demonstrated that hADSCs/AHAM transplantation into damaged uterine cavity could significantly increase vascular density of the rat endometrial tissue [103]. They proposed that the ability of hADSCs/AHAM to repair damaged endometrium may be related to the accelerated angiogenesis, where the expression of angiogenic factors in hADSCs was up-regulated. Zhao et al. [104] have extracted exosomes from ADSCs (ADSC-exo) and applied them to an IUA rat model, and found that ADSC-exo could maintain the normal structure of uterus while improve the endometrial regeneration and reproductivity. They proposed that local application of ADSC-exo in uterus as a novel strategy for the treatment of IUA and infertility. Researchers have recently study have injected autologous ADSCs combined with ShakeGel™3D directly into the mice uterus to repair the damaged endometrium and restore the fertility by activating the BMP7-Smad5 signaling pathway [105].

## Embryonic stem cells (ESCs)

Embryonic stem cells (ESCs) are derived from the blastocyst phase of the early mammal embryo. Compared with other stem cells, ESCs are truly pluripotent cells as they have originated from the endoderm of embryo and can differentiate into various cell types in special culture medium [62, 63]. ESCs have retained a normal karyotype, with high telomerase activity and significant long-term proliferation potential [62]. Such cells have shown promise for the treatment of various diseases including spinal cord injury [106], arrhythmia [107], liver injury [108], diabetes [109], cartilage repair [110], etc. However, so far few have reported application of the ESCs for the treatment of IUA.

In 2015, Yu et al. [111] co-cultured hESCs with mouse endometrial stromal cells to induce differentiation of hESCs into endometrioid epithelium, and their results showed that the expression levels of cytokeratin, epithelial cell adhesion molecule (EpCAM), ER, and PR in the co-culture group were significantly increased on the 21st day after induced differentiation, confirming that hESCs could be differentiated into endometrioid cells [111]. Song et al. [112] also co-cultured hESCs with endometrial stromal cells to induce endometrioid cells, and seeded the hESC-derived cells onto collagen scaffolds and transplanted them into a rat model for severe uterine damage. 12 weeks later, hESC-derived cells were observed to survive and have recovered the structure and function of uterine horn.

Nevertheless, the use of embryo-isolate stem cells has remained to be ethically controversial. Clinical trials have also been debated that in vitro induction of the hESCs can have the risk of tumorigenesis. Therefore, the application of ESCs in the treatment of IUA still has a long way to go.

## Amniotic membrane stem cells (AMSCs)

Amniotic membrane stem cells (ASCs), including amniotic mesenchymal stromal cells (AMSCs) and amniotic epithelial cells (AECs), are stem cell-like cells isolated from the mesenchymal and epithelial layers of the amniotic membrane from discarded amniotic tissue, which are readily available and abundant, with relatively fewer ethical concerns [64, 65].

AMSCs possess potential therapeutic features such as inflammatory suppression, angiogenesis promotion, anti-oxidative stress, and other beneficial properties. They have even shown an immunomodulatory capacity by paracrine action [65–69]. In recent years, AMSCs have been recognized as a suitable alternative source of seed cells for tissue engineering. Gan et al. [113] discovered that transplantation of hAMSCs could lower the level of messenger RNA of pro-inflammatory cytokines while increase that of anti-inflammatory cytokines, which implied that AMSCs can promote endometrial regeneration by immunomodulatory effects.

AECs are embryonic stem cell-like lineages with the capability of differentiation and adult stem cell-like immunomodulatory properties [114]. AECs have shown be effective for the treatment of lung fibrosis [115], brain injury [116], kidney injury [117], and hepatic fibrosis [118]. Recently, researchers have explored therapeutic potential of AECs on IUA. Li et al. [119] found that intraperitoneal injection of hAECs into IUA rats could alleviate fibrogenic progression, increase vascular density and restore the structure of uterine cavity. They also found that in vitro co-culturing hAECs with H2O2-damaged human endometrial mesenchymal stem cells (hEnSCs) could activate autophagy of hEnSCs through paracrine pathways. In 2019, Lai et al. [120] had filed a patent for AECs, which signified that AECs could be used to prevent IUA secondary to endometrium injury, repair the endometrial morphology in a mouse model for IUA, promote endometrial angiogenesis and mesenchymal cell proliferation, and improve the fertility of mice following uterine cavity injury.

## Limitation of stem cell therapy

Stem cell therapy holds a great promise for uterine repair and regeneration. However, despite the remarkable achievements made in the research, their clinical applications still face challenges, as most studies have been conducted on animal models with non-standardized study design. Variables of the treatment such as cell source, treatment time, cell number and injection method need to be notarized. The safety of such therapy also needs to be carefully assessed.

The preservation and clinical use of stem cells are both challenging. Current clinical trials have mainly used freshly thawed cell stocks [42]. However, cryopreservation and thawing may affect the viability and functionalities of the stem cells [121, 122]. Cryopreservation can cause reversible and irreversible cryoinjuries to the stem cells, leading to host T-cell cytolysis, and affect the survival, distribution and immunosuppressive properties of exogenous stem cells [121, 123]. MSCs are not intrinsically immune-privileged and their transplants may induce immune rejection. Allogeneic MSCs may induce a memory T-cell response under certain conditions, resulting in rejection of allogeneic stem cells [124, 125]. Furthermore, extensive in vitro expansion of stem cells may trigger replicative senescence, thereby affecting their therapeutic effectiveness [126].

**Fig. 3:**
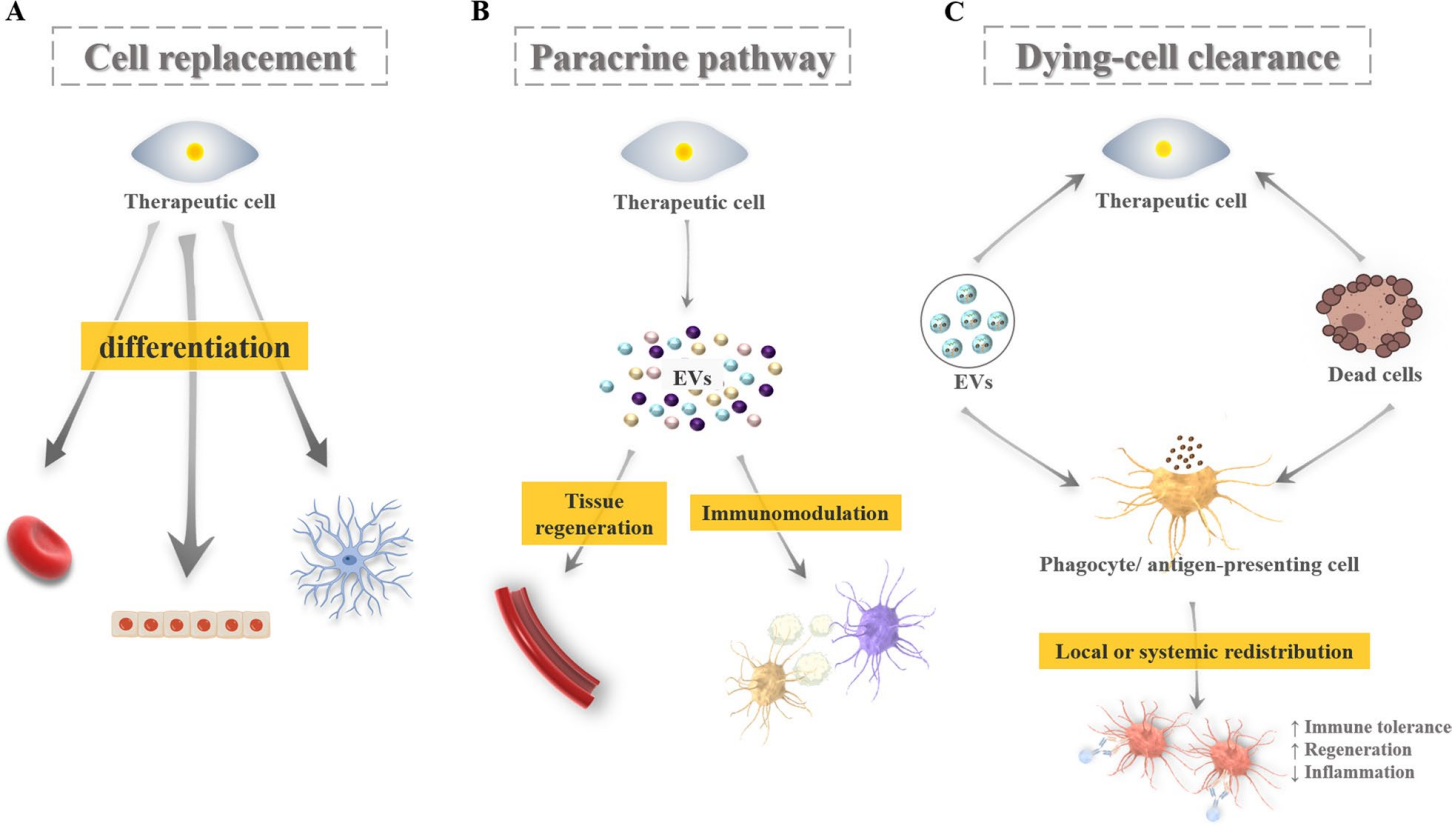
Potential mechanisms of stem cell-based therapy. **A** Cell replacement by stem cells multi-lineage differentiation. **B** Cell communication though paracrine signaling; **C**: Dying cell clearance through phagocytosis. Adapted from Wagoner and Zhao [128]

With regard to the mechanism of stem cell therapy, previous studies showed that it relied on the appropriate homing and engraftment capacity of stem cells [127]. To date, increasing evidence suggested that the key mechanism of stem cell therapy is related to their paracrine pathway rather than ability for differentiation. Stem cell-mediated paracrine factors may therefore overcome the limitations of cell-based therapy, though its effectiveness and safety need to be further validated. It has also been discovered recently that stem cells will die rapidly and be cleared by innate immune cells after transplantation, which suggested that reprogramming of the immune cells may enhance the therapeutic effect (Fig. 3) [128].

## Conclusion and future perspective

As a pervasively common postoperative complication of intrauterine surgery, IUA will inevitably affect the reproductive capability and full recovery of the endometrium, which constitute a vital role in reproduction and maternal health. Over the past a few decades, hysteroscopy, hormone therapy, and application of intrauterine devices have been tried to tackle IUA, but all showed some shortcomings. In recent years, exploitation of stem cell therapy to restore injured endometrium has become a promising new treatment approach. A growing number of animal experiments and clinical trials have focused on the effect and mechanisms with respect to stem cell therapy. Studies have shown that application of stem cells derived from bone marrow, endometrium, menstrual blood, adipose, embryo, and cord blood can facilitate restoration of the structure and function of the uterus. In addition, combination of stem cells with biopolymer materials such as scaffolds, hydrogel, and nanostructure lipid carrier, can improve its delivery and enhance the survival and therapeutic effect of transplanted cells. Stem cell-based therapy can promote the regeneration and repair of endometrium, disrupt formation of fibrosis, and promote regeneration of blood vessels through paracrine and immune modulations.

Nevertheless, there are also risks for stem cell-based therapy. The mechanism by which stem cells can promote endometrial regeneration is still unclear. In addition, immunogenicity and tumorigenicity should not be ignored, as previous context has stated that teratoma is a main obstacle to the clinical usage of stem cell-based therapies, in particular embryonic stem cells. Therefore, the selection of stable and safe stem cell types and transplantation methods requires more research.

In summary, the ability of stem cells to self-differentiate and distinct regeneration mechanism have made them an attractive candidate for treating gynecological diseases such as the IUA.

## Data Availability

Not applicable.

## Abbreviations

ADSCs: Adipose-derived stem cells
ADSC-exo: Exosomes from ADSCs
AECs: Amniotic epithelial cells
AHAM: Acellular human amniotic membrane
AMSCs: Amniotic mesenchymal stromal cells
AS: Asherman’s syndrome
ASCs: Amniotic membrane stem cells
BM-MSCs: Bone marrow mesenchymal stem cells
BMSCs: Bone marrow stem cells
CS: Collagen scaffold
EnSCs: Endometrial stem/progenitor cells
EnSCs-CM: EnSCs conditioned medium
EMA: The European Medicine Agency
eMSCs: Endometrial mesenchymal stem cells
EpCAM: Epithelial cell adhesion molecule
EPCs: Endothelial progenitor cells
ESCs: Embryonic stem cells
ESP: Endometrial side population
EVs: Extracellular vesicles
G-CSF: Granulocyte-colony stimulating factor
GFP: Green fluorescent protein
HA: Hyaluronic acid
hEnSCs: Human endometrial mesenchymal stem cells
HSG: Hysterosalpingography
hUCMSCs-EV: EV derived from hUC-MSCs
HUVECs: Human umbilical vein endothelial cells
IUA: Intrauterine adhesion
IUD: Intrauterine device
LIF: Leukemic inhibition factor
MSCs: Mesenchymal stem/stromal cells
PGS: Poly (glycerol sebacate)
PRP: Platelet-rich plasma
SP: Side population
TCRA: Transcervical resection of the adhesions
UC-MSCs: Umbilical cord-derived mesenchymal stromal cells
